# Spectral Analysis of the Bounded Linear Operator in the Reproducing Kernel Space *W*_2_^*m*^(*D*)

**DOI:** 10.1155/2014/691461

**Published:** 2014-08-28

**Authors:** Lihua Guo, Songsong Li, Boying Wu, Dazhi Zhang

**Affiliations:** ^1^Department of Mathematics, Harbin Institute of Technology, Harbin 150001, China; ^2^School of Management, Harbin Institute of Technology, Harbin 150001, China

## Abstract

We first introduce some related definitions of the bounded linear operator *L* in the reproducing kernel space *W*_2_^*m*^(*D*). Then we show spectral analysis of *L* and derive several property theorems.

## 1. Introduction

It is well known that spectral analysis of linear operators [1] is an important topic in functional analysis. For example, the matrix eigenvalue in linear algebra and eigenvalue problems for differential equation have been discussed emphatically. Two major reasons are as follows. Firstly, spectral analysis arises from vibration frequency problems and the stability theory of system. Secondly, spectral analysis comes from the need of discussing the structure of the operator and solving the corresponding equation by using the eigenvalue and spectral theorem. Also, spectral analysis can be used to study the structure of the solution for homogeneous or nonhomogeneous differential system and the normalized form of matrix which can be obtained clearly by matrix eigenvalues.

So far, the spectral decomposition method [2] has become a central topic in the theory of spectral analysis of linear operators. This method has been successfully applied in Hilbert space and perfect spectral decomposition theorem [3]. In recent years, the spectral decomposition method has been developed into spectral theorems of spectral operators and decomposable operators in Banach space [4, 5].

To our knowledge, reproducing kernel space has been applied in many fields, such as linear systems [6–8], nonlinear systems [9–11], operator equation, stochastic processes, wavelet transform, signal analysis, and pattern recognition [12–17]. Since the reproducing kernel space is a Hilbert space, this paper will apply the theory of spectral analysis for linear operator in the reproducing kernel space *W*_2_^*m*^(*D*) and derive some useful conclusions.

The paper is organized as follows. In Section 2, we introduce some related definitions for the eigenvalue of the bounded linear operator *L* in the reproducing kernel space *W*_2_^*m*^(*D*). In Section 3, the regular point and the spectral point of bounded linear operator *L* in *W*_2_^*m*^(*D*) are given. In Section 4, we show spectral analysis of the bounded linear operator *L* and also establish several theorems.  Section 5 ends this paper with a brief conclusion.

## 2. Related Definitions

**Definition 1 .**

Let *D* be an abstract set, *W*_2_^*m*^(*D*) the reproducing kernel space, and *BL*[*W*_2_^*m*^(*D*) → *W*_2_^*n*^(*D*)] the bounded linear operator space. ∀*L* ∈ *BL*[*W*_2_^*m*^(*D*) → *W*_2_^*n*^(*D*)] with *m*, *n* ∈ *N*, if there exists nonvanishing vector *u* ∈ *W*_2_^*m*^(*D*), such that

(1) $$\begin{array}{c} Lu=\lambda u\;\text{or}\;\;\left(\lambda I-L\right)u=0. \end{array}$$

Then *λ* is called an eigenvalue of *L* and *u* is called the eigenvector of *L* according to *λ*, where *I* denotes the identity operator.

**Definition 2 .**

∀*L* ∈ *BL*[*W*_2_^*m*^(*D*) → *W*_2_^*n*^(*D*)] and all eigenvectors and zero vector of *L* compose the eigenvector space which is denoted by *E*_*λ*_.

Obviously, *E*_*λ*_ is a linear closed subspace of *W*_2_^*m*^(*D*).

**Definition 3 .**

Denote the dimension of *E*_*λ*_ by dim⁡⁡*E*_*λ*_; it is called the multiplicity of eigenvalue *λ*. That is, dim⁡⁡*E*_*λ*_ is the number of vectors of maximum linear independence.

**Example 4 .**

Let *K*(*s*, *t*) be a binary function on *D* = {(*s*, *t*)∣*a* ≤ *s* ≤ *b*, *a* ≤ *t* ≤ *b*}, *L* ∈ *BL*[*W*_2_^*m*^(*D*) → *W*_2_^*n*^(*D*)], *m*, *n* ∈ *N* with

(2) $$\begin{array}{c} Lu\left(s\right)=\overset{b}{\underset{a}{\int }}K\left(s,t\right)u\left(t\right)dt,\;u\in W_{2}^{m}\left(D\right); \end{array}$$

then *λ* is the eigenvalue of *L* if and only if the following integral equation has nonzero solution:

(3) $$\begin{array}{c} \lambda u\left(s\right)-\overset{b}{\underset{a}{\int }}K\left(s,t\right)u\left(t\right)dt=0. \end{array}$$

If *K*(*s*, *t*) = ∑_*i*=1_^*n*^ *f*_*i*_(*s*)*g*_*i*_(*t*), {*f*_*i*_}_*i*=1_^*n*^ is a linear independence vector system, then (3) can be converted into the equivalent equation

(4) $$\begin{array}{c} \lambda u\left(s\right)-\overset{n}{\underset{i=1}{\sum }}f_{i}\left(s\right)\overset{b}{\underset{a}{\int }}g_{i}\left(t\right)u\left(t\right)dt=0. \end{array}$$

Thus we have the following results.

(a) If *λ* = 0, then (4) has nonzero solution if and only if *u* ∈ *W*_2_^*m*^(*D*) and ∫_*a*_^*b*^ *g*_*i*_(*t*)*u*(*t*)*dt* = 0, *i* = 1,2,…, *n*. It follows that, for the eigenvalue *λ* = 0 of *L*, the eigenvector space is infinite-dimensional.
(b) If *λ* ≠ 0, then the solution of (4) can be denoted by
  (5) $$\begin{array}{c} u\left(s\right)=\overset{n}{\underset{i=1}{\sum }}C_{i}f_{i}\left(s\right), \end{array}$$
  where *C*_*i*_ (*i* = 1,2,…, *n*) are constants.

Combine (5) with (4) and, in view of the linear independence of {*f*_*i*_}_*i*=1_^*n*^ in (5), *C*_*i*_ must satisfy the following linear equation system:

(6) $$\begin{array}{c} \overset{n}{\underset{j=1}{\sum }}C_{j}\overset{b}{\underset{a}{\int }}g_{i}\left(t\right)f_{j}\left(t\right)dt=\lambda C_{i},\;i=1,2,\ldots ,n. \end{array}$$

Summing up the above results, we can see that eigenvalues of (3) and (5) are equivalent, where *C*_*i*_ (*i* = 1,2,…, *n*) are undetermined coefficients. In addition, in order to solve the eigenvector, we just need to solve *C*_*i*_ (*i* = 1,2,…, *n*) in (5).

## 3. Regular Point and Spectral Point

In Definitions 1–3, we introduce the eigenvalue, eigenvector, eigenvector space, and dim⁡⁡*E*_*λ*_ of *L* for the homogeneous equation (1). However, for many problems in mathematics and physics, we just need to solve the following nonhomogeneous equation:

(7) $$\begin{array}{c} \left(\lambda I-L\right)u=f, \end{array}$$

where *L* is a given operator, *f* is a given vector, and *u* is an unknown vector. In order to discuss this problem, we need to introduce the following definitions and theorems.

**Definition 5 .**

Let *L* ∈ *BL*[*W*_2_^*m*^(*D*) → *W*_2_^*n*^(*D*)], *m*, *n* ∈ *N*, *D*(*L*)⊆*W*_2_^*m*^(*D*), and *R*(*L*)⊆*W*_2_^*n*^(*D*), where *D*(*L*) denotes the domain of *L* and *R*(*L*) denotes the range of values of *L*. If the inverse operator *L* ^−1^ of *L* exists and is linearly bounded, then *L* is called a regular operator.

Let *L* be a linear operator;  *D*(*L*)⊆*W*_2_^*m*^(*D*) and *R*(*L*)⊆*W*_2_^*n*^(*D*); if *L* ^−1^ exists, then *L* ^−1^ *L* = *I*_*D*(*L*)_ and *LL* ^−1^ = *I*_*R*(*L*)_, where *I*_*D*(*L*)_ and *I*_*R*(*L*)_ are, respectively, identity operators of subspace *D*(*L*) and *R*(*L*). Inversely, if there exists a linear operator *C* : *W*_2_^*n*^(*D*) → *W*_2_^*m*^(*D*), such that *CL* = *I*_*D*(*L*)_ and *LC* = *I*_*D*(*C*)_, then *L* ^−1^ exists and *I* ^−1^ = *C*. In fact, ∀*u*_1_, *u*_2_ ∈ *D*(*L*); if *Lu*_1_ = *Lu*_2_, then *u*_1_ = *CL* *u*_1_ = *CL* *u*_2_ = *u*_2_. Hence, *L* is invertible. Since *LC* = *I*_*D*(*C*)_, then ∀*v* ∈ *D*(*C*); we have *u* = *Cv* such that *Lu* = *v*. That is, *D*(*C*)⊆*R*(*L*).

Summing up the above disscusion, *R*(*L*) = *D*(*C*). Hence, we have *L* ^−1^ = (*CL*)*L* ^−1^ = *C* *LL* ^−1^ = *C*. Particularly, when *D*(*C*) = *W*_2_^*n*^(*D*) and *C* is a bounded linear operator, we can derive the following results.

**Theorem 6 .**

Let *L* ∈ *BL*[*W*_2_^*m*^(*D*) → *W*_2_^*n*^(*D*)], *m*, *n* ∈ *N*; then *L* is a regular operator if and only if ∃*C* ∈ *BL*[*W*_2_^*n*^(*D*) → *W*_2_^*m*^(*D*)], such that *CL* = *I*_*D*(*L*)_ and *LC* = *I*_*D*(*C*)_.

**Theorem 7 .**

Let *L* ∈ *BL*[*W*_2_^*m*^(*D*) → *W*_2_^*n*^(*D*)], *m*, *n* ∈ *N*; if *L* is a regular operator, then L* is also a regular operator and (L*)^−1^ = (*L* ^−1^)*.

**Proof**

Since *L* ^−1^ ∈ *BL*[*W*_2_^*n*^(*D*) → *W*_2_^*m*^(*D*)], *m*, *n* ∈ *N*, and

(8) $$\begin{array}{c} LL^{-1}=I_{W_{2}^{n}(D)},\;\;L^{-1}L=I_{W_{2}^{m}(D)}, \end{array}$$

by taking conjugate on both sides of the above formulas, we obtain

(9) $$\begin{array}{c} L^{*}{\left(L^{-1}\right)}^{*}=I_{W_{2}^{n^{*}}(D)},\;\;{\left(L^{-1}\right)}^{*}L^{*}=I_{W_{2}^{m^{*}}(D)}. \end{array}$$

In view of Theorem 6, we can see that L* is a regular operator and (L*)^−1^ = (*L* ^−1^)*. The proof is complete.

**Definition 8 .**

Let *D*(*L*)⊆*W*_2_^*m*^(*D*), *m* ∈ *N*, *λ* ∈ *C*; *C* denotes the complex number field.

1. *λI* − *L* is a regular operator, that is, *λI* − *L* is a one-to-one linear operator from *D*(*L*) to *W*_2_^*m*^(*D*). In addition, the inverse operator (*λI* − *L*)^−1^ is a linear bounded operator. Then *λ* is called a regular point of *L*. All regular points compose the regular set of *L*, which is denoted by *ρ*(*L*).
2. If *λ* is not a regular point, then *λ* is called a spectral point of *L*. All spectral points compose the spectral set of *L*, which is denoted by *σ*(*L*).

In view of Definition 8, we have *ρ*(*L*)⋃*σ*(*L*) = *C*. Then we have the following property results.

**Lemma 9 .**

Let *L* be a bounded linear operator in reproducing kernel space *W*_2_^*m*^(*D*), *m* ∈ *N*; then *λ* is a regular point of *L* if and only if ∀*f* ∈ *W*_2_^*m*^(*D*), there exists a solution *g* of (*λI* − *L*)*g* = *f*, which satisfies ||*g*|| ≤ *m*||*f*||, where m is a positive constant.

**Proof**

⇒ Since *R*(*λI* − *L*) = *W*_2_^*m*^(*D*), then ∀*f* ∈ *W*_2_^*m*^(*D*), ∃*g* ∈ *W*_2_^*m*^(*D*) such that (*λI* − *L*)*g* = *f*. In addition, in view of the boundedness of (*λI* − *L*)^−1^ and the Cauchy-Schwartz inequality, we have

(10) $$\begin{array}{c} ||g||=||{\left(\lambda I-L\right)}^{-1}f||\leq ||{\left(\lambda I-L\right)}^{-1}||||f||. \end{array}$$

Let *m* = ||(*λI*−*L*)^−1^|| > 0; then ||*g*|| ≤ *m*||*f*||.

⇐ Since (*λI* − *L*)*g* = *f*, we have *R*(*λI* − *L*) = *W*_2_^*m*^(*D*). Next, we will prove that *λI* − *L* is one-to-one. In fact, ∀*f* ∈ *W*_2_^*m*^(*D*); if (*λI* − *L*)*g*_1_ = *f*, (*λI* − *L*)*g*_2_ = *f*, then

(11) $$\begin{array}{c} \left(\lambda I-L\right)\left(g_{1}-g_{2}\right)=0; \end{array}$$

namely, the image of *g*_1_ − *g*_2_ is 0. Hence, ||*g*_1_ − *g*_2_|| ≤ *m*||0||; that is, *g*_1_ = *g*_2_. Therefore, *λI* − *L* is one-to-one and (*λI* − *L*)^−1^ exists. Furthermore, since ||*g*|| ≤ *m*||*f*||, we have

(12) $$\begin{array}{c} ||{\left(\lambda I-L\right)}^{-1}f||\leq m||f||; \end{array}$$

that is,

(13) $$\begin{array}{c} ||{\left(\lambda I-L\right)}^{-1}||\leq m. \end{array}$$

Hence, (*λI* − *L*)^−1^ exists and is a bounded linear operator. Summing up the above, *λI* − *L* is a regular operator, where *λ* is a regular point of *L*.

Lemma 9 shows that ∀*f* ∈ *W*_2_^*m*^(*D*); when *L* is a continuous linear operator and *λ* is the regular point of *L*, (*λI* − *L*)*g* = *f* has a unique solution *g*. Furthermore, the continuity of *g* depends on the right term. In other words, if {*f*_*i*_}_*i*=1_^*n*^ are column vectors and *f*_*n*_ → *f*, then *g*_*n*_ → *g*.

**Lemma 10 .**

Let *L* be a bounded linear operator in the reproducing kernel space *W*_2_^*m*^(*D*), *m* ∈ *N*. If *λ* is not the eigenvalue of *L* and (*λI* − *L*)*g*_1_ = (*λI* − *L*)*g*_2_, one has *L*(*g*_1_ − *g*_2_) = *λ*(*g*_1_ − *g*_2_), *g*_1_ = *g*_2_. That is, *λI* − *L* is invertible.

**Proof**

Otherwise, the invertible operator can convert the nonvanishing vector to nonvanishing vector. Hence, there exists *g* ≠ 0 such that (*λI* − *L*)*g* = 0. That is, *λ* is not the eigenvalue of *L*.

When *W*_2_^*m*^(*D*) is a finite dimension space and *λ* is not the eigenvalue of *L*, we can derive that *C* = *λI* − *L* is an invertible mapping. Obviously, *R*(*C*) = *W*_2_^*m*^(*D*). In fact, let {*e*_*i*_}_*i*=1_^*n*^ be the basis of *W*_2_^*m*^(*D*); then {(*λI* − *L*)*e*_*i*_}_*i*=1_^*n*^ is a linear independent system in *W*_2_^*n*^(*D*) and also a basis of *W*_2_^*n*^(*D*). Therefore, *R*(*L*) = *W*_2_^*n*^(*D*). In view of the inverse operator Theorem, (*λI* − *L*)^−1^ is bounded. It follows that *λ* ∈ *ρ*(*L*).

So, the proof of the theorem is complete.

Lemma 10 shows that regular point and spectral point are absolutely opposite for finite dimension normed spaces. That is, spectral point of *L* can only be an eigenvalue in finite dimension normed space. This is entirely consistent with the conclusion of the theory of linear algebra. But if *W*_2_^*m*^(*D*) is an infinite-dimensional space and *λ* is not the eigenvalue of *L*, then *λ* may not be a regular point of *L*, so far as *λI* − *L* is not a map from *W*_2_^*m*^(*D*) to *W*_2_^*n*^(*D*).

For example, let

(14) $$\begin{array}{c} Lu=\overset{b}{\underset{a}{\int }}u\left(t\right)dt,\;u\in W_{2}^{m}\left(D\right); \end{array}$$

∀*λ* ∈ *C*, ∫_*a*_^*b*^ *u*(*t*)*dt* = *λu*(*t*) has only zero solutions. Hence, *L* has not eigenvalue. That is, zero is not the eigenvalue. However, the range of values is all functions of the from ∫_*a*_^*b*^ *u*(*t*)*dt* for (0*I* − *L*). This shows that the spectral point is complex in infinite-dimensional space for the operator *L*.

Now, we will classify the spectral set by three situations.

- If *λI* − *L* is not one-to-one, then *λ* is called point spectral of *L*; the set of point spectral is denoted by *σ*_*p*_(*L*).
- If *λI* − *L* is one-to-one and *R*(*λI* − *L*) is dense in *W*_2_^*m*^(*D*), then *λ* is called continuous spectral of *L*; the set of continuous spectral is denoted by *σ*_*c*_(*L*).
- If *λI* − *L* is one-to-one and *R*(*λI* − *L*) is not dense in *W*_2_^*m*^(*D*), then *λ* is called residual spectral of *L*; the set of residual spectral is denoted by *σ*_*r*_(*L*).

Obviously, *σ*_*p*_(*L*), *σ*_*c*_(*L*), and *σ*_*r*_(*L*) are mutually disjoint sets and *σ*(*L*) = *σ*_*p*_(*L*) ∪ *σ*_*c*_(*L*) ∪ *σ*_*r*_(*L*).

## 4. Spectral Analysis

Let *L* ∈ *BL*[*W*_2_^*m*^(*D*) → *W*_2_^*n*^(*D*)], *m*, *n* ∈ *N*, $r=\lim_{n\to \infty }\sqrt[{n}]{||L^{n}||}$, ∀*ε* > 0, ∃*N* ∈ N*, ∀*n* > *N*, such that $\sqrt[{n}]{||L^{n}||}<r+\varepsilon <1$; that is, ||*L* ^*n*^||<(*r* + *ε*)^*n*^. In view of the completeness of *W*_2_^*m*^(*D*), there exists *m* > *N*, such that

(15) ||∑n=m∞Ln||≤∑n=m∞||Ln||≤∑n=m∞(r+ε)n=(r+ε)m(1−r−ε)−1.

Hence, ∑_*n*=0_^*∞*^ *L* ^*n*^ converges in the sense of ||·|| and the limit is denoted by *C* = ∑_*n*=0_^*∞*^ *L* ^*n*^.

Let *C*_*m*_ = ∑_*n*=0_^*m*^ *L* ^*n*^; then

(16) $$\begin{array}{c} C_{m}\left(I-L\right)=\left(I-L\right)C_{m}=I-L^{m+1}. \end{array}$$

For ||*C*_*m*_ − *C*|| → 0, *m* ≥ *N*, we have

(17) $$\begin{array}{c} ||L^{m+1}||\leq {\left(r+\varepsilon \right)}^{m+1}\to 0. \end{array}$$

If *m* → *∞*, then

(18) $$\begin{array}{c} C\left(I-L\right)=\left(I-L\right)C=I. \end{array}$$

Namely, 1 ∈ *ρ*(*L*) and (*I*−*L*)^−1^ = ∑_*n*=0_^*∞*^ *L* ^*n*^.

For ||*L*|| < 1, one obtains

(19) $$\begin{array}{c} ||{\left(I-L\right)}^{-1}||=||C||\leq \overset{\infty }{\underset{n=0}{\sum }}||L^{n}||=\frac{1}{1-||L||}. \end{array}$$

Summing up the above parts, we have the following theorems.

**Theorem 11 .**

Let *L* ∈ *BL*[*W*_2_^*m*^(*D*) → *W*_2_^*n*^(*D*)], *m*, *n* ∈ *N*, then one has the following.

1. Consider 1 ∈ *ρ*(*L*).
2. Consider (*I* − *L*)^−1^ = ∑_*n*=0_^*∞*^ *L* ^*n*^.
3. When ||*L*|| < 1, ||(*I*−*L*)^−1^|| ≤ 1/(1 − ||*L*||).

**Theorem 12 .**

Let *L* ∈ *BL*[*W*_2_^*m*^(*D*) → *W*_2_^*n*^(*D*)], *m*, *n* ∈ *N*; if $r=\lim_{n\to \infty }\sqrt[{n}]{||L^{n}||}$, then one has the following.

1. |*λ*| > *r* if and only if *λ* is a regular point of *L*.
2. When |*λ*| > *r*, (*λI*−*L*)^−1^ = ∑_*n*=0_^*∞*^(*L* ^*n*^/*λ* ^*n*+1^).
3. When |*λ*| > ||*L*||, ||(*λI*−*L*)^−1^|| ≤ (|*λ*|−||*L*||)^−1^.

**Proof**

∀*λ* ≠ 0, since (*λI* − *L*) = *λ*(*I* − *L*/*λ*), *λ* ∈ *ρ*(*L*) if and only if 1 ∈ *ρ*(*L*/*λ*). Replacing *L* by *L*/*λ* in Theorem 11, we have

(20) $$\begin{array}{c} \underset{n\to \infty }{\lim}\sqrt[{n}]{||\frac{L^{n}}{\lambda^{n}}||}=\frac{1}{\left|\lambda \right|}\underset{n\to \infty }{\lim}\sqrt[{n}]{||L^{n}||}<1; \end{array}$$

namely,

(21) $$\begin{array}{c} \left|\lambda \right|>\underset{n\to \infty }{\lim}\sqrt[{n}]{||L^{n}||}=r,\;1\in \rho \left(\frac{L}{\lambda }\right). \end{array}$$

Furthermore, we have

(22) $$\begin{array}{c} {\left(I-\frac{L}{\lambda }\right)}^{-1}=\overset{\infty }{\underset{n=0}{\sum }}{\left(\frac{L}{\lambda }\right)}^{n}=\overset{\infty }{\underset{n=0}{\sum }}\frac{L^{n}}{\lambda^{n}}. \end{array}$$

Then we obtain

(23) $$\begin{array}{c} {\left(\lambda I-L\right)}^{-1}=\frac{1}{\lambda }{\left(I-\frac{L}{\lambda }\right)}^{-1}=\overset{\infty }{\underset{n=0}{\sum }}\frac{L^{n}}{\lambda^{n+1}}. \end{array}$$

It follows that *λ* is a regular point of *L* and ||(*λI*−*L*)^−1^|| ≤ (|*λ*|−||*L*||)^−1^ with |*λ*| > *r*.

In addition, when |*λ*| > ||*L*||, we have ||*L*/*λ*|| < 1. In view of (2) of Theorem 11, we have

(24) $$\begin{array}{c} ||{\left(\lambda I-L\right)}^{-1}||<\frac{1}{\left|\lambda \right|}{\left(1-||\frac{L}{\lambda }||\right)}^{-1}={\left(\left|\lambda \right|-||L||\right)}^{-1}. \end{array}$$

The proof is complete.

**Theorem 13 .**

Let *L* ∈ *BL*[*W*_2_^*m*^(*D*) → *W*_2_^*n*^(*D*)], *m*, *n* ∈ *N*; then one has the following.

1. *ρ*(*L*) is an open set.
2. When *ρ*(*L*) is nonempty, ∀*λ*_0_ ∈ *ρ*(*L*); if $r_{\lambda_{0}}=\lim_{n\to \infty }\sqrt[{n}]{||{\left(\lambda_{0}I-L\right)}^{-n}||}$, then *λ* is a regular point of *L* and (*λI*−*L*)^−1^ = ∑_*n*=0_^*∞*^(−1)^*n*^(*λ*_0_ *I*−*L*)^−(*n*+1)^(*λ*−*λ*_0_)^*n*^, where |*λ* − *λ*_0_| < 1/*r*_*λ*_0__.
3. *σ*(*L*) is a closed set.
4. Consider $\sup_{\lambda \in \sigma (L)}\left|\lambda \right|\leq \lim_{n\to \infty }\sqrt[{n}]{||L^{n}||}$.

**Proof**

(1) If *ρ*(*L*) = *∅*, the conclusion is obvious. If *ρ*(*L*) ≠ *∅*, then

(25) λI−L=(λ−λ0)I+(λ0I−L)=[I+(λ−λ0)(λ0I−L)−1](λ0I−L),

where (*λ*_0_ *I*−*L*)^−1^ is a bounded linear operator in the reproducing kernel space *W*_2_^*m*^(*D*). We use (*λ* − *λ*_0_)(*λ*_0_ *I*−*L*)^−1^ instead of *L* in Theorem 11, such that

(26) $$\begin{array}{c} \underset{n\to \infty }{\lim}\sqrt[{n}]{||{\left[-\lambda \left(\lambda -\lambda_{0}\right){\left(\lambda_{0}I-L\right)}^{-1}\right]}^{n}||}<1. \end{array}$$

That is, when |*λ* − *λ*_0_| < 1/*r*_*λ*_0__, [*I*+(*λ*−*λ*_0_)(*λ*_0_ *I*−*L*)^−1^]^−1^ exists and is bounded. Hence, when |*λ* − *λ*_0_| < 1/*r*_*λ*_0__, *λ* ∈ *ρ*(*L*); that is, *ρ*(*L*) is an open set.

(2) If *ρ*(*L*) is nonempty, ∀*λ*_0_ ∈ *ρ*(*L*), let $r_{\lambda_{0}}=\lim_{n\to \infty }\sqrt[{n}]{||{\left(\lambda_{0}I-L\right)}^{-n}||}$. In view of (2) of Theorem 11 and (1) of Theorem 13, we have

(27) (λI−L)−1=(λ0I−L)−1[I+(λ−λ0)(λ0I−L)−1]−1=∑n=0∞(−1)n(λ0I−L)−(n+1)(λ−λ0)n.

(3) Since *ρ*(*L*)⋃*σ*(*L*) = *C* and (1), *σ*(*L*) is a closed set.

(4) In view of (1) of Theorem 12 and $r=\lim_{n\to \infty }\sqrt[{n}]{||L^{n}||}$, we have *σ*(*L*)⊆{*λ*∣|*λ*| ≤ *r*}, which means that $\sup_{\lambda \in \sigma (L)}\left|\lambda \right|\leq \lim_{n\to \infty }\sqrt[{n}]{||L^{n}||}$.

The proof is complete.

**Definition 14 .**

Let *L* ∈ *BL*[*W*_2_^*m*^(*D*) → *W*_2_^*n*^(*D*)], *m*, *n* ∈ *N*, *r*(*L*) = max⁡_*λ*∈*σ*(*L*)_⁡|*λ*|; *r*(*L*) is called the spectral radius of *L*.

From the purpose of solving equations, spectral radius has the following meanings.

1. For |*λ*| > *r*(*L*), due to the fact that *λ* is a regular point of *L*, then for any *f* ∈ *W*_2_^*m*^(*D*), (*λI* − *L*)*g* = *f* has a unique solution *g*.
2. For |*λ*| ≤ *r*(*L*), it cannot guarantee this equation has a solution for any *f* ∈ *W*_2_^*m*^(*D*). In many practical problems, in order to calculate the spectral range, one needs to estimate the spectral radius. In terms of (4) of Theorem 13, we can get *r*(*L*) ≤ ||*L*||. In practical terms, this estimate is convenient, but it is imprecise.

**Theorem 15 .**

Let *L* ∈ *BL*[*W*_2_^*m*^(*D*) → *W*_2_^*n*^(*D*)], *m*, *n* ∈ *N*; then $r(L)=\sup_{\lambda \in \sigma (L)}\left|\lambda \right|=\lim_{n\to \infty }\sqrt[{n}]{||L^{n}||}$.

**Proof**

In terms of (4) of Theorem 13, $r(L)\leq \lim_{n\to \infty }\sqrt[{n}]{||L^{n}||}$. Hence, one only needs to prove that $r(L)\geq \lim_{n\to \infty }\sqrt[{n}]{||L^{n}||}$. For |*λ*| > ||*L*||, one obtains

(28) $$\begin{array}{c} {\left(\lambda I-L\right)}^{-1}=\overset{\infty }{\underset{n=0}{\sum }}\frac{L^{n}}{\lambda^{n+1}}. \end{array}$$

Consider ∀*f* ∈ *W*_2_^*m*^(*D*), *f*((*λI*−*L*)^−1^) = ∑_*n*=0_^*∞*^(*f*(*L* ^*n*^)/*λ* ^*n*+1^). If |*λ*| > *r*(*L*), then *λ* is a regular point of *L*. In addition, since {*λ*∣|*λ*| > *r*(*L*)}, then Laurent expansions of *f*((*λI*−*L*)^−1^) are established, where |*λ*| > *r*(*L*).

Let *a* = *r*(*L*), ∀*ε* > 0; we have

(29) $$\begin{array}{c} \overset{\infty }{\underset{n=0}{\sum }}\frac{f\left(L_{n}\right)}{{\left(a+\varepsilon \right)}^{n+1}}<\infty . \end{array}$$

Let *B*_*n*_ = *L* ^*n*^/(*a*+*ε*)^*n*^, ∀*f* ∈ *W*_2_^m*^(*D*); then

(30) $$\begin{array}{c} \underset{n\geq 1}{\sup}\left|f\left(B_{n}\right)\right|<\infty . \end{array}$$

In terms of the resonance Theorem, {*B*_*n*_} must be bounded. It follows that there exists a positive constant *M*, such that ||*B*_*n*_|| ≤ *M* and ||*L* ^*n*^|| ≤ (*a*+*ε*)^*n*^||*B* ^*n*^|| ≤ (*a*+*ε*)^*n*^ *M*. Namely,

(31) $$\begin{array}{c} \underset{n\to \infty }{\lim}\sqrt[{n}]{||L^{n}||}<a+\varepsilon . \end{array}$$

Let *ε* → 0; then

(32) $$\begin{array}{c} r\left(L\right)=a\geq \underset{n\to \infty }{\lim}\sqrt[{n}]{||L^{n}||}. \end{array}$$

The proof is complete.

**Theorem 16 .**

Let *L* ∈ *BL*[*W*_2_^*m*^(*D*) → *W*_2_^*n*^(*D*)], *m*, *n* ∈ *N*; then *σ*(*L*) ≠ *∅*.

**Proof**

If *σ*(*L*) = *∅*, in view of the properties of the reproducing kernel space, *W*_2_^*m*^(*D*)≠{0}; hence, *I* ≠ {0}, where the unit element is denoted by *I*. In terms of the functional extension Theorem, ∃*f* ∈ *W*_2_^m*^(*D*), such that *f*(*I*) ≠ 0. In addition, ∀*λ*_0_ ∈ *ρ*(*L*) and ∃*r*_*λ*_0__ ∈ *R* ^+^; when |*λ* − *λ*_0_| < 1/*r*_*λ*_0__, we have

(33) $$\begin{array}{c} {\left(\lambda I-L\right)}^{-1}=\overset{\infty }{\underset{n=0}{\sum }}{\left(-1\right)}^{n}{\left(\lambda_{0}I-L\right)}^{-n-1}{\left(\lambda -\lambda_{0}\right)}^{n}. \end{array}$$

Note that

(34) $$\begin{array}{c} f\left({\left(\lambda I-L\right)}^{-1}\right)=\overset{\infty }{\underset{n=0}{\sum }}{\left(-1\right)}^{n}f\left({\left(\lambda_{0}I-L\right)}^{-n-1}\right){\left(\lambda -\lambda_{0}\right)}^{n}, \end{array}$$

in terms of the assumption that *σ*(*L*) = *∅* and Theorem 15; when |*λ*| > ||*L*||, one obtains

(35) $$\begin{array}{c} f\left({\left(\lambda I-L\right)}^{-1}\right)=\overset{\infty }{\underset{n=0}{\sum }}\frac{f\left(L^{n}\right)}{\lambda^{n+1}}. \end{array}$$

Therefore, when |*λ*| ≥ ||*L*|| + 1, we have

(36) $$\begin{array}{c} \left|f\left({\left(\lambda I-L\right)}^{-1}\right)\right|\leq \overset{\infty }{\underset{n=0}{\sum }}||f||\frac{||L^{n}||}{{||\lambda ||}^{n+1}}\leq ||f||\frac{1}{\left|\lambda \right|-||L||}\leq ||f||. \end{array}$$

That is, *f*((*λI*−*L*)^−1^) is bounded. In terms of the Liouville Theorem, *f*((*λI*−*L*)^−1^) must be a constant, so we have *σ*(*L*) ≠ *∅*.

The proof is complete.

**Definition 17 .**

If *L* ∈ *BL*[*W*_2_^*m*^(*D*) → *W*_2_^*n*^(*D*)], *m*, *n* ∈ *N*, $\lim_{n\to \infty }\sqrt[{n}]{||L^{n}||}=0$, then *L* is called a generalized nilpotent operator.

Definition 17 is the finite-dimensional space concept nilpotent operator in the infinite-dimensional space to promote. In the spectral theory of operators, generalized nilpotent operator is a kind of important operator.

In terms of Theorem 16 and the spectral radius theorem, one can obtain that the generalized nilpotent operator has only a spectral point 0. For example, let *L* ∈ *BL*[*W*_2_^*m*^(*D*) → *W*_2_^*n*^(*D*)], *m*, *n* ∈ *N*, [*a*, *t*]⊆*D*,

(37) $$\begin{array}{c} \left(Lu\right)\left(t\right)=\overset{t}{\underset{a}{\int }}u\left(\mu \right)d\mu ,\;u\in W_{2}^{m}\left(D\right). \end{array}$$

In terms of the property of *L*, one obtains

(38) $$\begin{array}{c} L^{n}u=\overset{t}{\underset{a}{\int }}\overset{t_{1}}{\underset{a}{\int }}\cdots \overset{t_{n-1}}{\underset{a}{\int }}u\left(\mu \right)d\mu dt_{n-1}\cdots dt_{1}. \end{array}$$

Note that

(39) $$\begin{array}{c} \left|\left(L^{n}u\right)\left(t\right)\right|\leq ||u||\overset{t}{\underset{a}{\int }}\overset{t_{1}}{\underset{a}{\int }}\cdots \overset{t_{n-1}}{\underset{a}{\int }}u\left(\mu \right)d\mu dt_{n-1}\cdots dt_{1}; \end{array}$$

we have

(40) $$\begin{array}{c} ||L^{n}u||\leq \frac{1}{n!}{\left(b-a\right)}^{n}||u||,\;u\in W_{2}^{m}\left(D\right). \end{array}$$

This shows that *L* is a generalized nilpotent operator; spectral point *λ* = 0 is not the eigenvalue of *L*.

**Definition 18 .**

Let *L* ∈ *BL*[*W*_2_^*m*^(*D*) → *W*_2_^*n*^(*D*)], *m*, *n* ∈ *N*, *λ* ∈ *C*; if there exists {*u*_*n*_}_*n*=1_^*∞*^ ∈ *W*_2_^*m*^(*D*), such that (*λI* − *L*)*u*_*n*_ → 0, then *λ* is called an approximate spectral point. All approximate spectral points are denoted by *σ*_*a*_(*L*); the other spectral point is called remainder spectral point. All the remainder spectral points are denoted by *σ*_*r*_(*L*).

**Theorem 19 .**

Let *L* ∈ *BL*[*W*_2_^*m*^(*D*) → *W*_2_^*n*^(*D*)], *m*, *n* ∈ *N*; then

1. *σ*_*p*_(*L*)⊆*σ*_*a*_(*L*),
2. *σ*_*a*_(*L*)⋂*σ*_*r*_(*L*) = *∅*, and *σ*_*a*_(*L*)⋃*σ*_*r*_(*L*) = *σ*(*L*),
3. *σ*_*r*_(*L*) is an open set,
4. ∂*σ*(*L*)⊆*σ*_*a*_(*L*), where ∂*σ*(*L*) denotes the boundary of *σ*(*L*),
5. *σ*_*a*_(*L*) is a nonempty closed set.

**Proof**

(1) If *λ* ∈ *σ*_*p*_(*L*), then there exists nonzero element *u* of *W*_2_^*m*^(*D*), such that

(41) $$\begin{array}{c} \left(L-\lambda I\right)u=0. \end{array}$$

Without loss of the generality, let ||*u*|| = 1; we choose *u*_*n*_ = *u*, *n* = 1,2,…, and then ||*u*_*n*_|| = 1 and (*L* − *λI*)*u*_*n*_ → 0; namely, *λ* ∈ *σ*_*a*_(*L*); this shows that *σ*_*p*_(*L*)⊆*σ*_*a*_(*L*).

(2) In terms of Definition 18, one obtains *σ*_*a*_(*L*)⋂*σ*_*r*_(*L*) = *∅* and *σ*_*a*_(*L*)⋃*σ*_*r*_(*L*) = *σ*(*L*).

(3) If *λ* ∈ *σ*_*r*_(*L*), then *λ* ∉ *σ*_*a*_(*L*). Hence, ∃*α* ∈ N*, such that

(42) $$\begin{array}{c} ||\left(L-\lambda I\right)u||\geq \alpha ||u||,\;u\in W_{2}^{m}\left(D\right). \end{array}$$

When |*λ*′ − *λ*| < *α*/2, ∀*u* ∈ *W*_2_^*m*^(*D*), we have

(43) $$\begin{array}{c} ||\left(L-\lambda^{\prime }I\right)u||\geq ||\left(L-\lambda I\right)u||-\left|\lambda -\lambda^{\prime }\right|\cdot ||u||\geq \frac{\alpha }{2}||u||. \end{array}$$

It shows that for any *λ*′ which satisfies |*λ*′ − *λ*| < *α*/2 it is impossible to be an approximate spectral point of *L*. Hence, if one can prove that when |*λ*′ − *λ*| < *α*/2, *λ*′ is not a regular point of *L*, then *λ*′ ∈ *σ*_*r*_(*L*). That is, *λ* is an inner point of *σ*_*r*_(*L*), so *σ*_*r*_(*L*) is an open set.

Now, we prove that when |*λ*′ − *λ*| < *α*/2, *λ*′ ∉ *ρ*(*L*). But not vice versa, ∃*λ*_0_ ∈ *C*, |*λ*_0_ − *λ*| < *α*/2; then *λ*_0_ ∈ *ρ*(*L*). Note that ||(*L* − *λ*′*I*)*u*|| ≥ ||(*L* − *λI*)*u*|| − |*λ* − *λ*′|||*u*|| ≥ (*α*/2)||*u*||; if *λ*′ = *λ*_0_, then

(44) $$\begin{array}{c} ||{\left(L-\lambda_{0}I\right)}^{-1}||<\frac{2}{\alpha }. \end{array}$$

In view of (2) of Theorem 13, let *μ* be a regular point of *L*; if |*μ* − *λ*_0_| < 1/*r*_*λ*_0__, then

(45) $$\begin{array}{c} r_{\lambda_{0}}=\underset{n\to \infty }{\lim}\sqrt[{n}]{||{\left(\lambda_{0}I-L\right)}^{-n}||}\leq ||{\left(\lambda_{0}I-L\right)}^{-1}||=\frac{2}{\alpha }. \end{array}$$

In a particular case, let *μ* = *λ*; note that

(46) $$\begin{array}{c} \left|\mu -\lambda_{0}\right|=\left|\lambda -\lambda_{0}\right|<\frac{\alpha }{2}<\frac{1}{r_{\lambda_{0}}}; \end{array}$$

then *λ* ∈ *ρ*(*L*). This is a contradiction with *λ* ∈ *σ*_*r*_(*L*). It follows that *σ*_*r*_(*L*) is an open set.

(4) Since *σ*(*L*) is a closed set, when *λ* ∈ ∂*σ*(*L*), *λ* ∈ *σ*(*L*). In addition, *λ* ∉ *σ*_*r*_(*L*), ∂*σ*(*L*)⊈*σ*_*r*_(*L*), so we have ∂*σ*(*L*)⊆*σ*_*a*_(*L*).

(5) Since *σ*_*a*_(*L*) = *σ*(*L*) − *σ*_*r*_(*L*), then *σ*_*a*_(*L*) is a closed set. Furthermore, since ∂*σ*(*L*)⊆*σ*_*a*_(*L*), *σ*_*a*_(*L*) ≠ *∅*, this shows that ∂*σ*(*L*) and *σ*_*a*_(*L*) are all nonempty sets.

The proof is complete.

## 5. Conclusions

This paper first introduces the eigenvalue, eigenvector, eigenvector space, and dim⁡⁡*E*_*λ*_ of the bounded linear operator *L* in the reproducing kernel space *W*_2_^*m*^(*D*). Then we show some definitions and properties of the regular operator. The regular set and spectral set of bounded linear operator are also introduced. From the solvability of the equation, we show the spectral classification and give three conditions. Finally, we introduce the spectral analysis of the bounded linear operator *L*. It includes the definitions of spectral radius, nilpotent operator, approximate spectral point, and remainder spectral point. We also establish some property theorems of the bounded linear operator in the reproducing kernel space *W*_2_^*m*^(*D*).

## References

[1] Dowson HR (1971). *Spectral Theory of Linear Operators*.

[2] West TT (1966). The decomposition of Riesz operators. *Proceedings of the London Mathematical Society III*.

[3] Weidmann J (1980). *Linear Operators in Hilbert Spaces*.

[4] Cui JL, Hou JC (2002). The spectrally bounded linear maps on operator algebras. *Studia Mathematica*.

[5] Hou J, Hou S (2002). Linear maps on operator algebras that preserve elements annihilated by a polynomial. *Proceedings of the American Mathematical Society*.

[6] Aronszajn N (1950). Theory of reproducing kernels. *Transactions of the American Mathematical Society*.

[7] Saitoh S (1988). *Theory of Reproducing Kernels and Its Applications*.

[8] Saitoh S, Alpay D, Ball J, Ohsawa T (1999). *Reproducing Kernels and Their Applications*.

[9] Cui MG, Lin YZ (2009). *Nonlinear Numerical Analysis in the Reproducing Kernel*.

[10] Yao HM (2010). *Reproducing Kernel Method for the Solution of Nonlinear Hyperbolic Telegraph Equation with an Integral Condition*.

[11] Geng F, Cui M (2011). A novel method for nonlinear two-point boundary value problems: combination of ADM and RKM. *Applied Mathematics and Computation*.

[12] Wu C-P, Jiang R-Y (2012). A state space differential reproducing kernel method for the 3D analysis of FGM sandwich circular hollow cylinders with combinations of simply-supported and clamped edges. *Composite Structures*.

[13] Geng FZ, Li XM (2012). A new method for Riccati differential equations based on reproducing kernel and quasilinearization methods. *Abstract and Applied Analysis*.

[14] Yang L, Shen J, Wang Y (2012). The reproducing kernel method for solving the system of the linear Volterra integral equations with variable coefficients. *Journal of Computational and Applied Mathematics*.

[15] Yang L (2012). Asymptotic regularity and attractors of the reaction-diffusion equation with nonlinear boundary condition. *Nonlinear Analysis. Real World Applications*.

[16] Lin YZ, Niu J, Cui M (2012). A numerical solution to nonlinear second order three-point boundary value problems in the reproducing kernel space. *Applied Mathematics and Computation*.

[17] Niu J, Lin YZ, Zhang CP (2012). Approximate solution of nonlinear multi-point boundary value problem on the half-line. *Mathematical Modelling and Analysis*.

